# Situation and determinants of the infant and young child feeding (IYCF) indicators in Madagascar: analysis of the 2009 Demographic and Health Survey

**DOI:** 10.1186/s12889-017-4835-1

**Published:** 2017-10-16

**Authors:** Hasina Rakotomanana, Gail E. Gates, Deana Hildebrand, Barbara J. Stoecker

**Affiliations:** Department of Nutritional Sciences, Oklahoma State University, 301 Human Sciences, Stillwater, OK 74078 USA

**Keywords:** Child feeding, IYCF indicators, Madagascar, Stunting

## Abstract

**Background:**

Studies evaluating child feeding in Madagascar are scarce despite its importance in child growth during the first two years of life. This study assessed the associations between the WHO infant and young child feeding (IYCF) indicators and stunting and identified determinants of inappropriate child feeding practices.

**Methods:**

The most recent Demographic and Health Survey was used including a total of 1956 infants aged 0–23 months. Logistic regressions were performed for the association between IYCF indicators and stunting and for the determination of risk factors for inappropriate feeding practices.

**Results:**

The rates of initiation of breastfeeding within one hour after birth (77.2%), continued breastfeeding at one year (99.6%) and timely introduction of solid, semi-solid or soft foods at 6–8 months (88.3%) were high. Exclusive breastfeeding under 6 months (48.8%), attaining minimum dietary diversity (22.2%) and consumption of iron-rich foods (19.6%) were relatively low. Higher length-for-age was associated with achieving minimum dietary diversity (*p*<0.01). The other indicators assessed (early initiation of breastfeeding, exclusive breastfeeding under 6 months, timely introduction of complementary foods and consumption of iron-rich foods) were not associated with stunting. Infants born to mothers who had first given birth at an age younger than 19 were more likely not to be breastfed within one hour after birth, not to be exclusively breastfed and not to have the recommended dietary diversity. Infants whose mothers had low media exposure were at increased risk of being inappropriately fed. Low household wealth also was associated with higher odds of not meeting the minimum dietary diversity.

**Conclusions:**

Despite almost total continued breastfeeding at one year and early initiation of breastfeeding by more than three-quarter of mothers, minimum dietary diversity scores were still low, confirming the need for more effective programs for improving child feeding practices in Madagascar. Improving dietary diversity in children aged 6–23 months may help reduce stunting. The identified risk factors for inappropriate feeding practices could be used in directing future nutrition sensitive interventions.

## Background

Childhood malnutrition remains a public health challenge, especially in low and middle-income countries, as more than one third of the disease burden is attributed to maternal and child undernutrition [1]. Findings reporting that growth faltering occurs sooner than expected in infants confirm the importance of nutrition during the first two years of life to ensure optimal growth and development for children [2]. Aware that feeding practices directly impact nutritional status of infants and young children, the WHO validated a set of indicators to assess infant and child feeding practices across countries [3]. The eight core indicators consider both breastfeeding (breastfeeding within the first hour after birth, exclusive breastfeeding, continued breastfeeding at 1 year) and complementary feeding practices (introduction of complementary foods at 6–8 months, minimum dietary diversity, minimum meal frequency, minimum acceptable diet and consumption of iron-rich foods).

Generally suboptimal IYCF indicators are observed in countries with the highest burden of malnutrition. For example, the reported rates of exclusive breastfeeding under 6 months were relatively low in countries such as Ethiopia (43%), Zambia (51.4%), Bangladesh (36.1%) and India (42%) [4]. Inappropriate complementary feeding practices were also reported in Ethiopia and Zambia where the proportion of children aged 6–23 months given more than four food groups in a day were respectively 7.1% and 37.1% [5]. Lack of dietary diversity also was found in Uganda with 29.5% and India with 16% of young children receiving four or more food groups in a day [6].

Madagascar is one of the 20 countries with the highest burden of malnutrition [7]. According to the WHO data in 2004, 61% of infants were breastfed within the first hour of birth, 67% were exclusively breastfed under 6 months and 78% were introduced to complementary foods between 6 and 8 months, showing better infant feeding practices than the average of the least developed and Sub-Saharan countries [8]. Yet for complementary feeding indicators such as dietary diversity (31%) or minimum acceptable diet (25%), practices still need improvement [8].

Knowing the factors associated with inappropriate feeding behaviors is critical to design and implement effective nutrition interventions and to inform policies. Few studies have looked at the determinants of IYCF practices in developing countries. Maternal factors such as education, knowledge and work status are the commons determinants of feeding practices across countries. In Uganda, results showed that educated mothers were more likely to have appropriate complementary feeding practices regarding minimum meal frequency, dietary diversity, minimum acceptable diet and iron-rich food consumption [9]. Studies from Bangladesh [10] and Nepal [11] found similar results as non-educated mothers were less likely to meet the recommendations for complementary feeding. Also, maternal knowledge about IYCF was associated with higher dietary diversity score in Ethiopia [12] and mothers who had 4 or more prenatal visits were more likely to report feeding their children with the recommended dietary diversity [13]. Moreover, increased maternal access to financial resources was reported to have positive association with dietary diversity and minimum acceptable diet criteria in nine sub-Saharan African countries [9]. Children living in the poorest households were more likely to have untimely introduction to complementary foods in India and Pakistan [10]. Similar results were reported by using pooled data from Kenya, Tanzania and Uganda [12].

Household factors influenced feeding practices. Children living in families that grew fruits and vegetables and owned livestock had higher dietary diversity scores in Southern Ethiopia [13]. Likewise, a recent study from Kenya reported that higher agricultural biodiversity was associated with higher dietary diversity scores in children aged 24–59 months [14]. Area of residence also has been suggested to play a role in IYCF practices; in Sri Lanka children living in urban areas and in tea estates were less likely to be introduced to complementary food at 6–8 months than children in rural areas [15]. For Madagascar, the results of the Comprehensive Food and Nutrition Security and Vulnerability Analysis (CFSVA + N) showed that improved maternal education was associated with early initiation of breastfeeding and with achieving minimum dietary diversity and minimum acceptable diet [16]. The report also stated that family wealth was associated with having greater diet diversity in complementary foods.

Regarding the association between IYCF and child growth, study results are not consistent, especially when stunting is used as an indicator. Marriott et al. [17] used pooled data from 14 developing countries to determine the association between IYCF indicators and stunting in children under 2 years old. Only timely introduction of solid and semi-solid foods, having a minimum acceptable diet and consumption of iron-fortified foods were associated with lower risk of stunting. Meeting minimum dietary diversity guidelines was also associated with better length-for age z-scores (LAZ) in young children from Bangladesh and Zambia [4]. Continued breastfeeding at 1 year without appropriate complementary feeding practices was inversely correlated with HAZ in Zimbabwe, Ethiopia and Zambia [4, 5]. Additionally, in a study using the Cambodian Demographic and Health Survey (DHS) data, of the eight IYCF indicators only exclusive breastfeeding until 6 months of age was associated with lower rates of stunting [18]. Not all of the IYCF indicators are associated consistently with child anthropometrics and findings tend to be different according to the country setting. Thus, there is a need to study the relationship between child undernutrition and the IYCF indicators at the country level.

In the case of Madagascar, only a few studies have looked at the infant and young child feeding practices in relation to child nutrition. For instance, a study reported that infant complementary feeding indicators were correlated with total energy intake and adequacy of minimum diet diversity in infants aged 6–23 months in urban areas [19]. They also found that dietary diversity was positively associated with LAZ. Another study looked at the relevance of two designed cross-sectional and longitudinal infant and children feeding indicators and their relationship with LAZ in infants 6–17 months [20]. They elaborated cross sectional and longitudinal feeding indicator scores including breastfeeding status, bottle feeding, dietary diversity score, food group frequency score and feeding frequency score. They noticed that the cross-sectional indicators were not significantly associated with LAZ in contrast to the longitudinal indicators in which the score was obtained from the sum of the cross-sectional indicators.

Given the importance of child feeding in growth and development during the first two years of life and the few published studies on IYCF indicators in Madagascar, more data are needed to direct decision-making processes and future interventions. This study looked at the association of the WHO IYCF indicators and child stunting in Madagascar. We also aimed to investigate the maternal and household factors associated with inappropriate IYCF practices in order to have a better understanding of such practices at the national level. To our knowledge, our study is the first using nationwide data investigating the WHO child feeding indicators and associated factors as well as their association with stunting.

## Methods

Publicly available data from the latest Demographic and Health Survey (2009) in Madagascar were used. The DHS was designed to collect nationally representative information about fertility, family planning, maternal and child health, and nutrition [21]. A two-stage sampling method was used. The first stratum was the administrative divisions of Madagascar, called regions, then the population in each region was divided into urban-rural constituting the second stratum. Households were then randomly sampled within each stratum proportionally to the actual distribution of the population. For the purpose of this study, we used the child dataset containing information about 1956 children born within the 5 years immediately prior to the time of the survey and their mothers and households. From this dataset, we analyzed data from infants and young children aged 0–23 months.

Binary variables corresponding to each of the definitions of IYCF indicators were created in accordance with the WHO recommendations [3]. The minimum dietary diversity variable was created from the diet diversity score of each child and coded as “1” if the child had eaten at least from 4 or more food groups the day before the interview and as “0” if less than four food groups were consumed. The food groups were defined as 1) grains, roots and tubers, 2) legumes and nuts, 3) dairy products, 4) flesh foods, 5) eggs, 6) vitamin A-rich fruit and vegetables and 7) other fruit and vegetables. Information was not collected about the meal frequency; thus, we were not able to evaluate the indicators 6 and 7: minimum meal frequency and minimum acceptable diet. Because no data about the consumption of iron-fortified foods were collected, indicator 8: consumption of iron-rich foods was estimated based on the consumption of flesh foods (meat, poultry and liver/organ meats).

Stunting was defined by the 2006 WHO growth standards: children less than two years of age with LAZ below −2 were considered stunted [22], and a binary variable was created for LAZ.

Logistic regressions were used to determine the association between the IYCF indicators and stunting as well as to assess the relationship between selected maternal and household factors and IYCF indicators. All the statistical analyses were computed on SAS, v. 9.4 (SAS Institute, Cary, NC, USA) and a significance level of 5% was chosen.

## Results

### Characteristics of the infants and mothers in Madagascar

A total of 40.7% of the children aged 6–23 months were stunted (Table 1). More than half of the mothers (53.9%) were younger than 19 when they first gave birth. A substantial proportion of the mothers had no formal education (28.6%) and almost half of them only attended primary school (49.7%). Exposure to media was very low because most mothers never read newspapers nor magazines (84.9%), listened to radio (46.6%) nor watched television (85.9%). The great majority of the households lived in rural areas (82.2%). Nearly half (49.6%) of the children lived in households within the two lowest quintiles of the wealth index. Additionally, more than half of the households (57.6%) did not have toilets and only 35.4% utilized improved sources of drinking water such as piped water, protected wells and rainwater.

**Table 1:** Characteristics of the infants and young children aged 0–23 months and their mothers, Madagascar 2009 (*n* = 1956)

Variables	Percentage (%)
Child characteristics	
Sex	
-Male	49.7
-Female	50.3
Nutritional status	
-Not stunted	59.3
-Stunted	40.7
Maternal characteristics	
Age at first birth	
-Younger than 19	53.9
-19 and older	46.1
Maternal education	
-No education	28.6
-Primary	49.7
-Secondary	19.9
-Higher	1.8
Exposure to media	
Frequency of reading newspaper/magazine	
-Not at all	84.9
-Once a week	8.8
-More than once a week	3.9
-Almost every day	2.1
Frequency of listening to radio	
-Not at all	46.6
-Once a week	10.5
-More than once a week	11.2
-Almost every day	31.6
Frequency of watching TV	
-Not at all	85.9
-Once a week	2.7
-More than once a week	2.4
-Almost every day	8.9
Maternal working status	
-Working	88.6
-Not working	11.3
Household characteristics	
Wealth Index	
-Poorest	29.5
-Poor	20.1
-Middle	18.4
-Richer	16.0
-Richest	15.8
Area of residence	
-Rural	82.2
-Urban	17.7
Toilet facilities	
-No toilet	57.6
-Latrines (pit/hanging/bucket)	38.1
-Flush toilets	4.2
Drinking water source	
-Unimproved^a^	64.5
-Improved^b^	35.4

^a^Unimproved source of water: unprotected well and spring water, river

^b^Improved source of water: piped water, tube wells, protected wells, protected spring water

### Situation of IYCF indicators in Madagascar in 2009

**Fig. 1:**
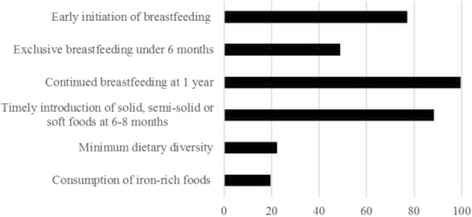
Achievement of infant and young child feeding (IYCF) indicators (%) for Madagascar based on the DHS 2009. Data were not available for minimum meal frequency and minimum acceptable diet

A substantial number of infants were put to the breast within the first hour after birth (77.2%) and continued breastfeeding at one year was almost universal (99.6%) as seen in Fig. 1. However, exclusive breastfeeding was relatively low as less than half of the mothers (48.8%) exclusively breastfed their infants during the first 6 months. A total of 88.3% of the infants received complementary foods at 6–8 months. Only 22.2% of the infants aged 6–23 months ate from four or more food groups. A small proportion (19.6%) reported eating an iron-rich food the previous day.

### Determinants of inappropriate IYCF in Madagascar

Because of the high compliance with continued breastfeeding at one year (only one observation did not comply), the indicator was not tested in regression models. Infants whose mothers gave birth for the first time at an age younger than 19 years were more likely not to have breastfed within the first hour. Also, the households with more resources (third and fourth quintiles) were more likely to have appropriate early initiation of breastfeeding practices (OR = 0.63 and OR = 0.58, respectively) compared to the poorest households (Table 2).

**Table 2:** Determinants of inappropriate infant and young child feeding (IYCF) practices, Madagascar 2009

Variables	Late introduction of breastfeeding (after 1 h)	Not exclusive breastfeeding under 6 months	Untimely introduction of semi-solid, solid and soft foods	Diet diversity score less than 4	Not consuming iron-rich foods
Child factor					
Sex (ref. male)	1.09 (0.88–1.35)	0.75 (0.52–1.09)	1.04 (0.50–2.18)	0.91 (0.70–1.20)	1.34 (1.04–1.73)^*^
Maternal factors					
Age at first birth					
-19 and older	1	1	1	1	1
-Less than 19	1.25 (1.01–1.54)^*^	1.59 (1.10–2.31)^*^	1.29 (0.60–2.76)	1.61 (1.26–2.05)^***^	1.41 (1.09–1.82)^***^
Maternal education					
-No formal education	1	1	1	1	1
-Primary	0.83 (0.65–1.06)	0.90 (0.58–1.38)	0.66 (0.29–1.51)	0.73 (0.52–1.02)	0.71 (0.49–1.02)
-Secondary	0.77 (0.56–1.05)	0.69 (0.40–1.18)	0.67 (0.22–2.01)	0.21 (0.15–0.30)^***^	0.19 (0.13–0.27)^***^
-Higher	1.23 (0.57–2.63)	4.21 (0.48–37.08)	1.92 (0.19–19.93)	0.04 (0.02–0.11)^***^	0.03 (0.01–0.08)^***^
Media exposure					
Reading newspaper					
-Not at all	1.23 (0.57–2.70)	1.04 (0.30–3.66)	0.91 (0.11–7.86)	16.00 (6.81–37.60)^***^	7.61 (3.67–15.79)^***^
-At least once a week	1.17 (0.51–2.68)	1.14 (0.30–4.33)	0.20 (0.01–3.66)	4.20 (1.73–10.24)^**^	1.89 (0.87–4.08)
-Almost every day	1	1	1	1	1
Listening to radio					
-Not at all	1.33 (1.04–1.70)^*^	1.21 (0.79–1.85)	1.69 (0.65–4.44)	2.77 (2.08–3.69)^***^	2.57 (1.91–3.44)^***^
-At least once a week	0.98 (0.72–1.34)	0.870(0.53–1.42)	2.23 (0.67–7.151)	1.26 (0.92–1.73)	1.64 (1.16–2.31)^**^
-Almost every day	1	1	1	1	1
Watching TV					
-Not at all	1.17 (0.79–1.71)	0.94 (0.48–1.84)	1.64 (0.37–7.34)	7.44 (5.12–10.81)^***^	9.28 (6.36–13.54)^***^
-At least once a week	0.83 (0.44–1.56)	1.02 (0.39–2.68)	1.44 (0.11–18.08)	2.71 (1.50–4.90)^**^	3.07 (1.68–5.59)^***^
-Almost every day	1	1	1	1	1
Work status					
-No	0.80 (0.56–1.13)	1.04 (0.62–1.74)	1.31 (0.47–3.70)	0.52 (0.36–0.74)^***^	0.37 (0.26–0.54)^***^
-Yes	1	1	1	1	1
Household factors					
Wealth Index					
-Poorest	1	1	1	1	1
-Poorer	0.83 (0.62–1.12)	1.28 (0.72–2.27)	1.28 (0.38–4.36)	0.95 (0.61–1.49)	1.13 (0.70–1.84)
-Middle	0.63 (0.46–0.87)^**^	1.19 (0.63–2.26)	1.00 (0.26–3.78)	0.58 (0.38–0.90)^*^	0.639 (0.41–0.99)^*^
-Richer	0.58 (0.41–0.82)^**^	0.81 (0.44–1.49)	1.58 (0.41–6.08)	0.36 (0.24–0.54)^***^	0.59 (0.37–0.93)^*^
-Richest	0.78 (0.56–1.08)	0.60 (0.31–1.15)	1.97 (0.51–7.61)	0.10 (0.07–0.12)^***^	0.09 (0.06–0.13)^***^
Area of residence					
-Urban	1	1	1	1	1
-Rural	1.09 (0.82–1.44)	1.07 (0.67–1.72)	1.58 (0.52–4.73)	4.00 (3.01–5.32)^***^	5.22 (3.90–6.99) ^***^

Results expressed as crude odds ratios OR (95% CI)

^*^ *P* < 0.05, ^**^ *P* < 0.01, ^***^ *P* < 0.001

Maternal age at first birth was the only factor significantly associated with exclusive breastfeeding under six months. Infants of mothers who first gave birth at an age younger than 19 years had 59% higher risk (OR = 1.59) of not being exclusively breastfed during the first 6 months.

For the complementary feeding indicators, none of the factors tested were associated with the time of introduction of solid, semi-solid or soft foods. Infants whose mothers attended primary school or higher had lower odds of having inadequate dietary diversity. Maternal exposure to media was also associated with adequate dietary diversity in infants. For instance, infants whose mothers did not listen to radio at all were more likely to have inadequate dietary diversity compared to those whose mothers listened to radio almost everyday. Likewise, infants were more likely to have inadequate dietary diversity if their mothers did not read newspaper or did not watch TV at all. Additionally, infants born to younger mothers had higher risks of being fed with inappropriate dietary diversity. Infants living in rural areas were also more likely to not have reached the minimum dietary diversity (OR = 4.00) compared to infants in urban areas. Infants of mothers who worked outside the home were at higher risk of having inadequate dietary diversity. The odds of having inappropriate dietary diversity decreased with increased wealth of the family.

Girls were more likely to not have been given iron-rich foods (OR = 1.34). Children whose mothers attended secondary school or higher education had lower chances of inadequate consumption of iron-rich foods. Infants born to mothers who do not read newspaper at all were more likely to not be given iron-rich foods. Infants whose mothers did not work outside the home and those living in a household with higher wealth were more likely to consume iron-rich foods. Finally, children living in rural areas had higher odds of having inadequate iron-rich food consumption.

### Stunting and IYCF

Only attaining the minimum diet diversity had a significant association with LAZ among the six IYCF indicators tested. Children with lower Z scores were less likely to have been given four or more food groups the day before the survey [OR = 0.92 (0.86–0.97), *p* < 0.01] (Data not shown). Because children’s weight was not reported in the 2009 DHS data for Madagascar, we could not test for association between IYCF practices and wasting (low length-for-weight) or underweight (low weight-for-age).

## Discussion

### IYCF indicators for Madagascar

For the breastfeeding indicators, our results suggest that although the rate of exclusive breastfeeding under 6 months was low, the indicators for two other breastfeeding practices, i.e. early initiation and continued breastfeeding, were high in Madagascar. A total of 77.2% of the children were put to the breast within one hour after birth and continued breastfeeding at one year was very high (99.6%). Compared to the data previously published by WHO [8], all of the breastfeeding indicators showed improvement except for the exclusive breastfeeding rate (67% in 2004 and 48.8% in 2009). In 2004, the WHO report did not communicate data on mothers working outside the home, but the difference in the reported rates of exclusive breastfeeding between 2004 and 2009 might be due to the increase in the number of working mothers since 2004. In 2009, a total of 87.5% of the mothers of infants aged 0–6 months were working outside the home, of which 51.6% did not practice exclusive breastfeeding. Because of time constraints and other logistic challenges, they were more likely to have practiced mixed feeding.

For the complementary feeding indicators, the rate of timely introduction of complementary foods from 2004 to 2009 also improved from 78% to 88.9%. A decrease from 31% to 22.2%was noticed in the percentage of infants who received the minimum dietary diversity between 2004 and 2009. For the DHS 2009, data collection was conducted from November 2008 to August 2009, which coincided with a political crisis in the country. Protestations against the then-government led to the resignation of the President and the rise of a transitional government. The transitional government was unrecognized by the international community, including donors and foreign governments, which sanctioned Madagascar by suspending their assistance programs [23]. As most of the public investment relevant to health and education relied on external funding, basic social services were diminished leaving the population very vulnerable, especially women and children [24]. In fact, the level of poverty was increased from 74.1% in 2005 to 81.8% in 2010 [25]. Because dietary diversity is closely linked to food access and availability [26], this might have contributed to the drop in the percentage of children achieving the minimum dietary diversity. Additionally, political instability is one of the basic causes of inadequate nutritional status in children [24]. In 2010, there were even fewer infants in rural areas who had received the minimum dietary diversity (14.1%) according to the CFSVA + N analysis [16].

### Stunting and IYCF

Only the minimum dietary diversity variable was positively associated with LAZ as children who had four or more food groups were more likely to have higher *z* scores, and thus would be less likely to be stunted. This outcome is concordant with the results found in the literature in different countries with high prevalence of stunting. Using pooled data from 14 low-income countries, Marriott et al. reported that attaining the minimum dietary diversity was associated with lower odds of stunting [17]. Also, studies using nationwide survey data from Bangladesh [27] and India [6] concluded that higher dietary diversity was associated with higher LAZ/HAZ. These findings highlight the importance of the quality of the diet for child growth especially during the first two years of life.

No significant association was found between the other three IYCF indicators (for which we had data) and stunting. Similar results were found in Cambodia, Haiti, Kenya and Uganda while using DHS data [4]. The only significant associations were between stunting and the introduction of solid, semi-solid or soft foods in Bangladesh and the minimum acceptable diet in Zimbabwe. The authors argued that the 24 h-recall nature of the indicators does not allow the investigator to capture the whole situation of feeding practices. Because the questions only consider the feeding practices of the previous day (the last 24 h), no matter how the infant had been fed in the past, as long as the caregivers had appropriate feeding practices the previous day, they will still be compliant with the indicator. The researchers suggested that the lack of sensitivity of the indicators might explain the lack of association between some IYCF indicators and child anthropometric measurements [4].

However, besides IYCF indicators there are multiple determinants of stunting, as it is a multifactorial problem. Rakotomanana et al. [28] found that increased age, more severe anemia level, being a male and decreased maternal height were associated with higher risk of stunting in infants aged 0–23 months in Madagascar. Also, infants currently being breastfed, infants living in households using iodized salt and in urban areas had lower odds of stunting. Region of residence was also a factor affecting stunting risk in the study.

### Determinants of inappropriate IYCF practices

Among the IYCF practices, sex was only a determinant for the consumption of iron-rich foods. No difference was seen in the consumption of flesh foods between sexes. However, there were more boys than girls being given organ meats even though the overall consumption meats was only 3.4% at 6–23 months.

Being born to a mother who gave birth at 19 or older or living in a family with somewhat more resources (in the third and fourth wealth quintile) was associated with an improved chance of initiating breastfeeding within the first hour after birth. A study using the Nigeria DHS reported that mothers with higher wealth were more likely to comply with the early initiation of breastfeeding [29]. More wealthy households may have more access to health care and may have been in contact with health care professionals who encouraged better breastfeeding practices.

Lower odds of inadequate diet diversity in infants were associated with higher maternal education, greater maternal exposure to media and household wealth. Similar results were found in Bangladesh [10], Uganda [9] and Ethiopia [13] as children of mothers with no formal education had higher odds of not meeting the minimum dietary diversity criteria. Regarding maternal exposure to media, a nationwide study in India [6] also reported that mothers who were less exposed to media might have less nutrition knowledge, as it is an important channel to spread nutrition information. In Southern Ethiopia, children had higher dietary diversity score when their mothers had received IYCF information on mass media in the last one month [13]. Additionally, studies across countries confirmed that increased family wealth was associated with better chances of meeting minimum dietary diversity [9, 15]. Higher household wealth generally means having access to more diverse food and more having more resources to be allocated to childcare and nutrition. Our results also showed that infants of working mothers had higher odds of inadequate dietary diversity. There is no clear explanation about the association between the maternal working status and the dietary diversity score except that working mothers generally spend time away from their children, which may lead to poor complementary feeding practices. Caretakers may be less knowledgeable about infant and young children nutrition and good feeding practices.

The consumption of iron-rich foods was associated with secondary or higher maternal education and higher wealth. Children living in rural areas were less likely to eat flesh foods. The overall poor dietary diversity and poor consumption of iron-rich food may be linked to access to food and to the general food insecurity in the country. Analyses from the CFSVA + N concluded that the seasonality of earnings due to a non-diversified income source in Malagasy households affects the access to food throughout the year [16]. Thus, 84% of the households experienced a time in the year when they did not have sufficient food. Yet, when food was available, other factors may play a role in inappropriate complementary feeding such as maternal or caretaker nutrition knowledge or attitudes [30].

### Implications for programs and nutrition interventions

Achieving feeding indicators appropriately has been associated with children’s anthropometric measurements in several countries [4]. Despite a few improvements in some IYCF indicators between 2004 and 2009 in Madagascar, efforts are still needed to improve breastfeeding and complementary feeding practices.

This study showed that several maternal and household characteristics are associated with inappropriate feeding practices in Madagascar. Younger maternal age (less than 19 y) at first birth was associated with sub-optimal breastfeeding, failure to achieve minimum dietary diversity and inadequate consumption of iron-rich foods. Sex education programs among youth and a specific focus on delaying early onset of first pregnancy may be needed as 53.3% of the Malagasy mothers in this sample were younger than 19 when they delivered their first child. Additionally, pregnant adolescents are more likely to give birth to low birthweight babies and are at risk of adverse birth outcomes [31].

Higher maternal education also has been linked to improved complementary feeding practices in other developing countries [9, 32]. Additionally, women’s empowerment interventions may lead to better nutritional outcomes. For instance, a study done across sub-Sahara African countries demonstrated that women who made decisions on large purchases and who had control over the household income were more likely to have adequate complementary feeding practices [33]. Thus, there is a need for nutrition-sensitive interventions in educational programs for women and girls to improve feeding practices in Madagascar. With the increasing number of mothers working outside home, these interventions should target other caretakers in the household as well such as grandmothers or elder siblings. Grandmothers’ knowledge regarding IYCF practices has been associated with better child feeding in Nepal [34].

Integrated nutrition interventions aiming to improve overall nutrition outcomes also have been shown to be efficient in ameliorating inadequate IYCF practices. A pilot study implementing the Essential Nutrition Actions (ENA) framework, was done in two districts of Madagascar in 2000, reported a significant increase in early breastfeeding, in exclusive breastfeeding under 6 months, and in meeting the minimum meal frequency for young children [35]. Through technical support and various trainings, as well as micronutrient supplementation, the ENA promotes key messages about maternal and child nutrition including feeding practices. The authors reported that involving a wide array of partners and creating a favorable policy environment through policy analysis and development were the key factors for the success of the pilot study. Thus, implementing such programs on a larger scale should be helpful in an effort to improve IYCF practices in Madagascar.

Despite being one of the few nationwide analyses of the IYCF indicators and associated factors in Madagascar, our study had limitations. Because of the lack of precision in the instruments during data collection, children’s weights were not reported for the 2009 DHS for Madagascar preventing us from looking at the association between wasting and underweight with the IYCF indicators. Additionally, although our study used the most recent nationwide data that includes nutrition information, the situation might have changed since 2009. This emphasizes the need for another nationwide representative survey to monitor the changes in the nutrition situation of the country.

## Conclusions

Several IYCF indicators for Madagascar were low, especially for the complementary feeding practices. Our results showed that children who have higher LAZ were more likely to have achieved the recommended dietary diversity. Younger maternal age at first birth, low maternal education and low household wealth were associated with inappropriate feeding practices. Addressing food insecurity, increasing access to food, preventing first pregnancy at a young age and creating income generation opportunities may be needed in order to implement programs that improve complementary feeding practices in Madagascar.
